# How vegetation in flows modifies the turbulent mixing and spreading of jets

**DOI:** 10.1038/s41598-017-05881-1

**Published:** 2017-07-26

**Authors:** Michele Mossa, Mouldi Ben Meftah, Francesca De Serio, Heidi M. Nepf

**Affiliations:** 1 Department of Civil, Environmental, Land, Building Engineering and Chemistry, Polytechnic University of Bari, Bari, Italy; 2 Department of Civil and Environmental Engineering, Massachusetts Institute of Technology, Cambridge, MA USA

## Abstract

While studies on vegetated channel flows have been developed in many research centers, studies on jets interacting with vegetation are still rare. This study presents and analyzes turbulent jets issued into an obstructed cross-flow, with emergent vegetation simulated with a regular array of cylinders. The paper presents estimates of the turbulence diffusion coefficients and the main turbulence variables of jets issued into a vegetated channel flow. The experimental results are compared with jets issued into unobstructed cross-flow. In the presence of the cylinder array, the turbulence length-scales in the streamwise and transverse directions were reduced, relative to the unobstructed crossflow. This contributed to a reduction in streamwise turbulent diffusion, relative to the unobstructed conditions. In contrast, the transverse turbulent diffusion was enhanced, despite the reduction in length-scale, due to enhanced turbulent intensity and the transverse deflection of flow around individual cylinders. Importantly, in the obstructed condition, the streamwise and transverse turbulent diffusion coefficients are of the same order of magnitude.

## Introduction

Aquatic vegetation provides a wide range of ecosystem services^1–3^. The uptake of nutrients and production of oxygen improve water quality^4^. The widespread planting in waterways could strongly contribute to the removal of nitrogen and phosphorous^5^. Seagrasses form the foundation of many food webs and vegetation promotes biodiversity by creating different habitats with spatial heterogeneity in the stream velocity^6^. Marshes and mangroves reduce coastal erosion by damping waves and storm surges^7^ and riparian vegetation enhances bank stability^8^. These services are all influenced in some way by the flow field existing within and around the vegetated region. At the same time, vegetation also affects flow structure and turbulence which in turn impact the transport of sediments and dissolved species. Therefore, vegetation is not just a static element of marine and fluvial ecosystems, unchanging with changing conditions, but it interacts with different processes at different scales, e.g. blade scale, patch scale or canopy scale^9–16^. Especially in this time of a changing climate, which could alter hydrological conditions, the monitoring of vegetation development is a fundamental activity in coastal and river monitoring and management^17–22^, both to protect ecological services and control flood and erosion risks.

Since the importance of flows through regions with vegetation has been recognized, many experimental and theoretical studies have been carried out to study many aspects of the interaction of channel flows and vegetation^3, 23–30^.

**Figure 1:**
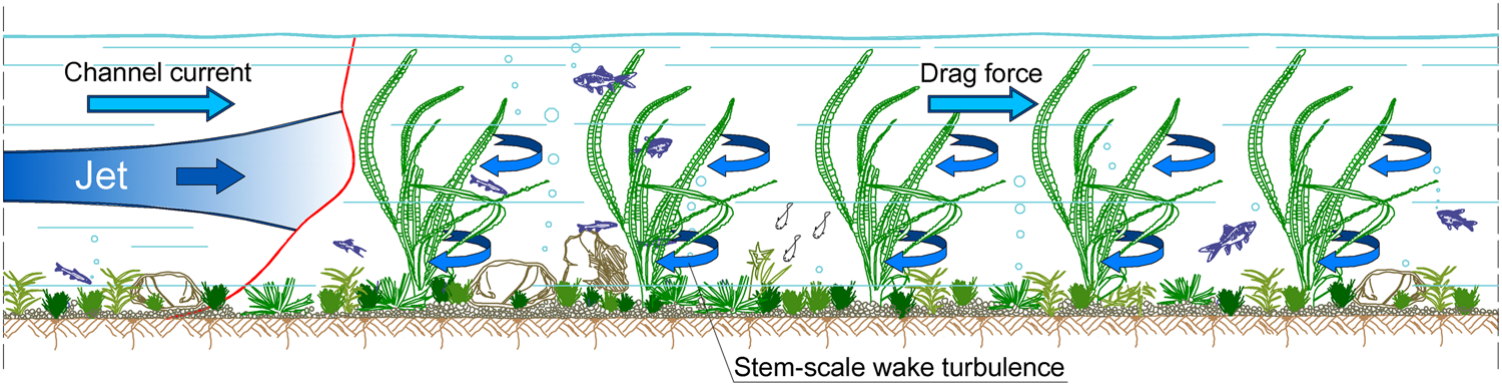
Example of patterns of a jet flow in a channel current with aquatic plants and stem scale wake turbulence.

Nevertheless, a further key point remains poorly investigated, that is the influence of vegetation on a turbulent jet, i.e. a discharged effluent^31^. Jet mixing has been extensively studied in the simpler case of unobstructed flows^32–36^, revealing the influence on jet evolution by the initial jet characteristics (e.g., nozzle shape, dimensions, flow rate), the boundary conditions (e.g., topography, bathymetry) and the hydrodynamic features of the ambient current. Even if detrainment is usually associated with buoyancy-driven flows, such as plumes or density currents flowing in a stratified environment, Mossa and De Serio^31^ proved theoretically that detrainment occurs also when a momentum-driven jet is issued in a not-stratified obstructed current. This finding is relevant, because it can be extended to unconventional ideas of jets through porous obstructions, such as the case of outflows from different sources spreading among oyster farms, wind farms, solar plants as well as aerial pesticides sprayed onto orchards or river jets flowing at mouths through bar deposits^37^ (see Appendix 1 for some examples). In the present paper the turbulent integral length scales, turbulent diffusion coefficients, and advective terms are analyzed in the streamwise and spanwise directions for a turbulent jet entering an obstructed flow and compared to the same jet in absence of vegetation. The flow obstructions, referred to as stems, are arranged as a regular array of emergent rigid cylinders. Finally, the energy dissipation rates of jets released in both unobstructed and obstructed flows are investigated. Turbulent energy production and dissipation are both closely related to the large-scale eddies^38^ responsible for mass and momentum exchange in channel flows, and these eddies are very different in unobstructed and obstructed flows. This study explains the typical mechanisms of turbulence spreading and energy production/dissipation for jets flowing in a vegetated current (Fig. 1).

### Theoretical approach

When jets interact with cylinders, the flow structure strongly depends on the relationship between the cylinder diameter, *d*, the distance between the stems, *s*, and the jet cross sectional length-scale, *b*. The key geometric parameters for a square array of cylinders are: the surface-to-surface distance between the cylinders *s*; the frontal area per unit array volume *a* = *nd*, with *n* the number of elements per unit planar area, and the solid volume fraction *ϕ* ≈ π*ad*/*4*, i.e., the volume within the array occupied by solid elements. For further details, see Appendix 1. Within the array the flow is spatially heterogeneous at the scale of the individual elements and often unsteady in time. To remove the temporal heterogeneity, the instantaneous equations of the vegetated current are averaged over a time longer than the time scale of turbulence or unsteadiness in the flow (denoted by an overbar). A spatial average^39, 40^ is not applied, because it would erase important information on the spatial variation along and transverse to the jet.

### Transport of tracers and turbulent kinetic energy

Hereafter we consider a rectangular array of stems located in a fluid of depth *H*. The model canopy is spatially uniform and emergent, i.e. its height is greater than or equal to the water depth. The ambient current is assumed uniform. The time-averaged turbulent transport of a solute concentration is described by the following equation

1 $$\frac{\partial \overline{c}}{\partial t}+\frac{\partial \overline{{u}_{i}c}}{\partial {x}_{i}}={K}_{ii}\frac{{\partial }^{2}\overline{c}}{\partial {x}_{i}^{2}}$$

where the overbar indicates the time-average operator and the prime symbol denotes the turbulent fluctuations, *c*(**x**) is the solute concentration, **v**(**x**) = (*u*, *v*, *w*) = (*v*_*1*_, *v*_*2*_, *v*_*3*_) is the fluid velocity, **x** = (*x*, *y*, *z*) = (*x*_*1*_, *x*_*2*_, *x*_*3*_), with *x* = *x*_*1*_, *y* = *x*_*2*_ and *z* = *x*_*3*_ the longitudinal, transversal and vertical axes, respectively, and *K*_*ii*_ are the coefficients for dispersion. For further details see Tanino and Nepf^30^.

In the analysis of the flow-dispersion interaction, the turbulent kinetic energy is important in determining the turbulent dispersion coefficient and thus the mass transport^30^. For high Reynolds numbers, assuming that the production term is of order of the dissipation term, the equation of the turbulent kinetic energy is

2 $$\frac{{\rm{\partial }}k}{{\rm{\partial }}t}+\frac{{\rm{\partial }}\bar{{u}_{i}}k}{{\rm{\partial }}{x}_{i}}={D}_{k}\frac{{{\rm{\partial }}}^{{\rm{2}}}k}{{\rm{\partial }}{x}_{i}^{{\rm{2}}}}$$

where $k=\frac{1}{2}\overline{{u}_{i}^{^{\prime} }{u}_{i}^{^{\prime} }}$ is the time-averaged turbulent kinetic energy and *D*_*k*_ is the turbulent diffusion coefficient, which can be expressed as the product of a length scale and a velocity scale. A physical meaningful velocity scale is $\sqrt{k}$. Consequently, we consider

3 $${D}_{k}=l\sqrt{k}$$

with *l* the integral length scale associated with turbulent eddies. Equation (2) is formally analogous to eq. (1) and, therefore, assuming that the Prandtl number is O(1), the cross-correlation between the time-averaged turbulent kinetic energy and the $\overline{u}$, $\overline{v}$ and $\overline{w}$ velocity components could be analyzed and related to the time-averaged solute concentration $\overline{c}$ transport by the mean flow $\overline{u}\overline{c}$, $\overline{v}\overline{c}$ and $\overline{w}\overline{c}$.

Furthermore, analogously to eq. (3), Tanino and Nepf^30^ assumed that the net dispersion coefficients of eq. (1) could be set equal to

4 $${K}_{ii}=\alpha \sqrt{k}{l}_{i}$$

where the scale factor *α* could be different for horizontal and vertical diffusion, even if generally it is of O(1).

In the present study, the integral length scale *l*_*i*_ is evaluated by multiplying the integral time scale *T*_*u*_ by the local time-averaged velocity $\overline{{u}_{i}({\bf{x}})}$, where *T*_*u*_ is estimated by the autocorrelation function of the turbulent velocity fluctuations (see Tanino and Nepf^30^ for a more complete description). In an unobstructed flow, *l* increases with the scale of the diffusing patch, until the largest length scale is reached, which is defined by the flow domain^41^. In the case of a jet entering an unobstructed current, it is reasonable to assume that the maximum value of the longitudinal mixing length scale *l*_*x*_ is O(*H*), where *H* is the channel flow depth, because the jet interacts with the channel current, which fills the flow depth. In contrast, the transversal mixing length scale is expected to be of O(*b*), where *b* is the length scale of the jet’s transverse cross section, since in this direction the ambient channel velocity (secondary current) is small compared to the longitudinal one.

Emergent canopies impose a specific structure on both the mean and turbulent flow over the entire water column. In flows with emergent vegetation, assuming that *H* is greater than *s* and *d*, the stems dissipate eddies with scales greater than the stem scales of *s* and *d*, while contributing additional turbulent energy at these stem scales. Thus, the dominant turbulent length scale within a canopy is shifted downward from the analogous condition without vegetation. In particular, in a channel with a regular array of cylinders, the integral length scale of turbulence is set by the smaller of the stem diameters, *d*, or the distance between the stems *s*, regardless of the water depth^30, 42^. In other words, for *d* ≤ *s*, turbulence is generated within stem wakes (if the Reynolds number is sufficient) so that *l* = *d*; on the contrary, for *d* > *s*, turbulence is generated within the pore channels so that *l* = *s*.

Finally, for low solid volume fractions (*ad* less than 0.01) the integral length scale of turbulence should have an intermediate value between the open channel and vegetated channel, i.e. *l* = O(min(*d*, *s*) to *H*). Further details can be found in literature^43–45^.

### Energy equation

The momentum equation in the longitudinal direction *x* for a plane compound jet with vegetation is

5 $$\overline{u}\frac{\partial \overline{u}}{\partial x}+\overline{v}\frac{\partial \overline{u}}{\partial y}=\frac{1}{\rho }\frac{\partial \overline{{\tau }_{t}}}{\partial y}-\frac{1}{\rho }\overline{{F}_{x}}$$

where $\overline{{\tau }_{t}}$ is the turbulent shear stress equal to $-\rho \overline{u^{\prime} v^{\prime} }$, and $\overline{{F}_{x}}$ is the vegetation drag force in the *x* direction, i.e. the resistance due to form and viscous drag over the stem (for further details, see Rajaratnam^46^ and Mossa and De Serio^31^).

Multiplying eq. (5) by $\rho \overline{u}$ and integrating it from *y* = 0, the center of jet, to $y=\overline{b}$, the outer boundary of the jet where $\overline{u}$ is close to the flow current velocity *U*_*e*_ (see Mossa and De Serio^31^), we get

6 $$\underset{0}{\overset{\bar{b}}{\int }}\rho {\bar{u}}^{2}\frac{{\rm{\partial }}\bar{u}}{{\rm{\partial }}x}dy+\underset{0}{\overset{\bar{b}}{\int }}\rho \bar{u}\bar{v}\frac{{\rm{\partial }}\bar{u}}{{\rm{\partial }}y}dy=\underset{0}{\overset{\bar{b}}{\int }}\bar{u}\frac{{\rm{\partial }}\bar{{\tau }_{t}}}{{\rm{\partial }}y}dy-\underset{0}{\overset{\bar{b}}{\int }}\bar{u}{\bar{F}}_{x}dy$$

Since the longitudinal time-averaged velocity is greater than the other components, the mean kinetic energy per unit volume is

7 $$\overline{E}=\frac{1}{2}\rho {\overline{u}}^{2}$$

and, therefore, eq. (6) becomes

8 $$\underset{0}{\overset{\bar{b}}{\int }}\frac{D\bar{E}}{Dt}dy=-\underset{0}{\overset{\bar{b}}{\int }}\bar{{\tau }_{t}}\frac{{\rm{\partial }}\bar{u}}{{\rm{\partial }}y}dy-\underset{0}{\overset{\bar{b}}{\int }}\frac{1}{2}\rho {C}_{D}a{\bar{u}}^{3}dy$$

which assumes a quadratic drag law for the vegetation drag force^47^ and *C*_*D*_ is the bulk drag coefficient (for details see Nepf^10^).

Equation (8) shows that the rate of decrease of the kinetic energy flux, i.e. the left side of the equation, is equal to the rate at which turbulence is produced by the Reynolds shear stress (first term of RHS) and the vegetation drag (second term of RHS). In vegetated channel flows (see Nepf^10^) the shear production is much smaller than the vegetation drag production due to the wakes of the cylinders, and, therefore, could be neglected. However, in the case of jets, where the shear production is larger than in channel flows, it is reasonable to consider that the first term of RHS of eq. (8) is smaller than the second term of RHS only at the interface of the jet with the channel flow and at large distance from the nozzle, where the jet is dispersed and the flow resembles that of a channel flow.

It is reasonable to assume that the turbulent kinetic energy budget is reduced to a balance between the viscous dissipation *ε* and the production *P*_*w*_. Therefore, it is possible to write

9 $$\varepsilon \approx {P}_{w}\Rightarrow {k}^{3/2}{l}^{-1}\approx \overline{u^{\prime} v^{\prime} }\frac{\partial \overline{u}}{\partial y}+\frac{1}{2}{C}_{D}a{\overline{u}}^{3}$$

since ε scales with *k* and *l*, and $\overline{u}$ can be scaled on a relevant velocity scale. Equation (9) states that, apart from Reynolds shear stress, the production of turbulent kinetic energy within the model array is due to the wake turbulence generation by the cylinders, which will scale mainly with *C*_*D*_, *a* and $\overline{u}$. Even if eq. (9) has been obtained for the case of a compound jet, which is mathematically simpler to treat, it is reasonable to conclude that the same conclusions are valid for the most complex configuration of jets issued into a crossflow (see Nepf^10^).

## Method

### Experimental procedure

**Figure 2:**
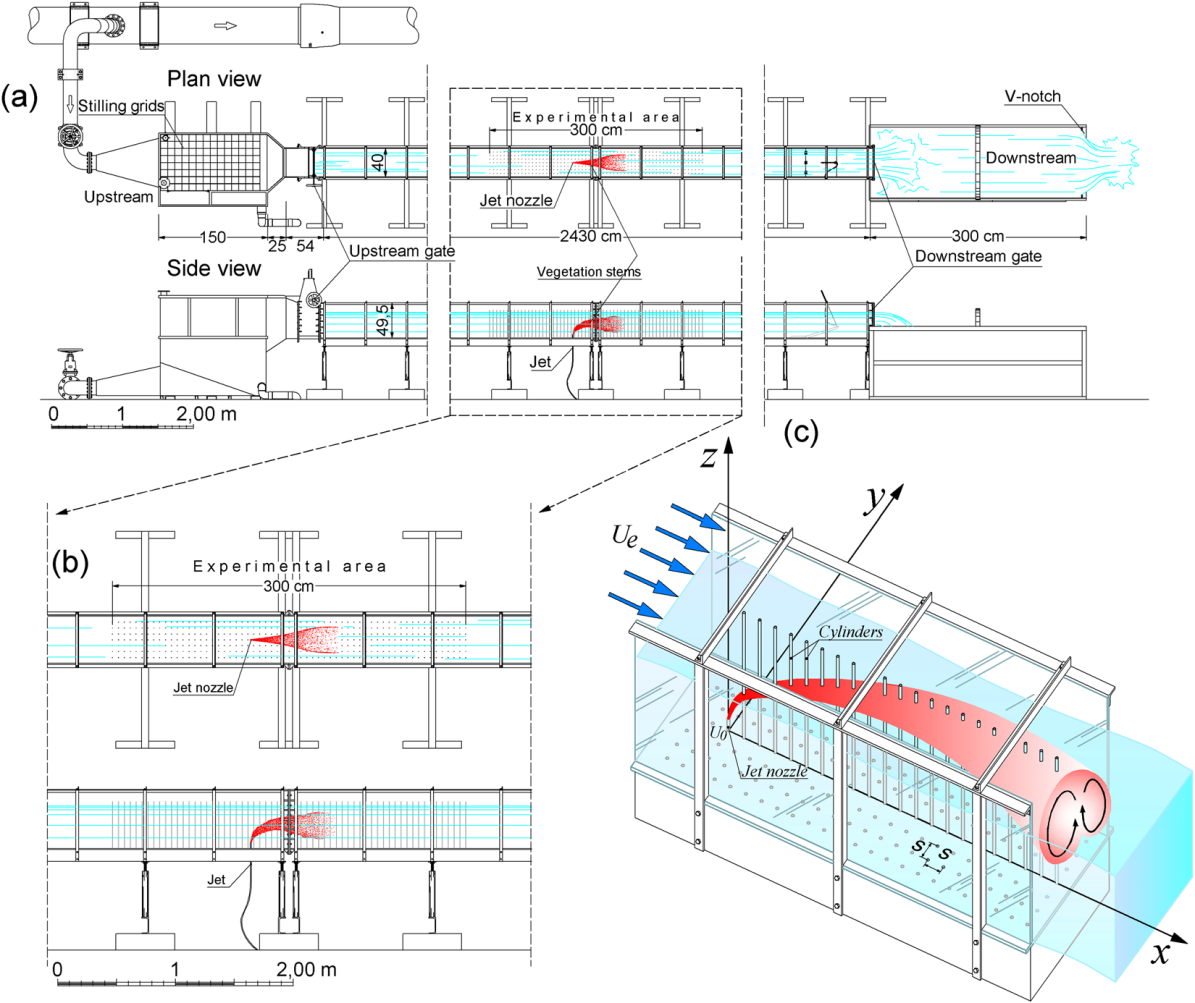
(**a**) Plan and side views of the channel; (**b**) close-up of the experimental area; (**c**) sketch of the jet with the rigid cylinder array (for the sake of clarity some stems are shown only with their projections on the channel bottom) and the coordinate axes.

The experimental runs were carried out in a smooth rectangular channel, with horizontal bed, which is extensively described in Ben Meftah *et al*.^13^. The channel was 25.0 m long, 0.40 m wide and 0.50 m deep (Fig. 2). The lateral walls and the bottom surface of the channel were made of Plexiglas.

A square array of rigid circular steel cylinders was used to simulate vegetation stems. The stem diameter, *d*, was equal to 0.003 m. The stems were inserted into a plywood board, which was 3.0 m long, 0.398 m wide and 0.02 m thick, which in turn was fixed along the channel bottom. In order to reduce the effect of plywood board thickness on the experimental area, two other 3.0 m x 0.398 m x 0.02 m plywood boards, without vegetation stems, were attached to the channel bottom at both the upstream and the downstream ends of this area. Stems were spaced longitudinally and transversally with the same distance *s* 0.05 m, so that the stem density, *n*, was 400 stems/m^2^, and the projected plant area per unit volume, was *a* = *nd* = *dH*/*s* ^*2*^ *H* = *d*/*s* ^*2*^ = 1.2 m^−1^, with *ϕ* = *nπd* ^*2*^/*4* = 0.00283.

The jet source with constant discharge was placed at the center of the experimental area, 15.0 m and 0.2 m from the inlet and the side-walls of the channel, respectively. It consisted of a circular metallic pipe with a diameter, *D* of 0.003 m. The jet-nozzle axis was vertical. The vertical distance from the channel bottom surface to the jet nozzle was equal to 0.03 m. We defined *x* = 0, *y* = 0 and *z* = 0.03 m as the Cartesian coordinates at the jet nozzle center, with *x*, *y* and *z* coordinates denoting the longitudinal, lateral and vertical directions, respectively (Fig. 2). For further details, see Ben Meftah *et al*.^13^.

The three components of instantaneous velocity (*u*, *v*, *w*) were sampled at 25 Hz using a 3D Nortek Acoustic Doppler Velocimeter (ADV), using a velocity range setting of ±0.30 m/s, which had a velocity accuracy of ±1%. The sampling volume was 27 mm^3^. The procedure of ADV despiking/filtering described by Goring and Nikora^48^ was used when spikes in the data sequence were detected.

In order to understand the cylinder effects on the jet behavior, two sets of experiments were conducted. The first investigated the jet discharged into an unobstructed channel with cross-flow (runs U1 to U4), and the second investigated the same jet discharged into an obstructed cross flow (runs O1 to O4). The main characteristics of all runs are described in Table 1, where *U*_*0*_ is the initial jet velocity, *U*_*e*_ is the cross-flow velocity, *R* = *U*_*0*_/*U*_*e*_ is the initial jet to crossflow velocity ratio, *Re* is the channel Reynolds number and *Re*_*0*_ is the initial jet Reynolds number.

**Table 1:** Main parameters of the experimental runs.

Flow type	Runs	*H* [cm]	*U*_*e*_ [ms^−1^]	*U*_*0*_ [ms^−1^]	*R* [−]	Re [−]	Re_0_ [−]
Jet in an unobstructed flow	U1	37	0.16	5.90	37.36	16036	13845
	U2	30	0.19	5.90	30.29	20383	15437
	U3	37	0.16	3.93	24.91	18802	10822
	U4	30	0.19	3.93	20.20	20733	10468
Jet in an obstructed flow	O1	37	0.16	5.90	37.36	23054	19904
	O2	30	0.19	5.90	30.29	26282	19904
	O3	37	0.16	3.93	24.91	24591	14154
	O4	30	0.19	3.93	20.20	26282	13270

For the unobstructed runs, measurements of velocity were made in the plane of flow symmetry *xz* with *y*/*D* = 0. For jets discharged into the obstructed cross flow, the array somewhat restricted the ADV placement, so that the velocity measurements were taken along the plane parallel to the plane of flow symmetry at *y*/*D* = 8.34. Extensive measurements were also made in the transversal planes.

## Results and Discussion

In the present paragraph, the results of the jets in a crossflow with and without cylinders are presented, considering that at further distance from the nozzle the jet behaves as a compound jet theoretically analyzed by Mossa and De Serio^31^. As shown in Table 1 four configurations have been analyzed. For the sake of brevity, only the results of one jet configuration without cylinders and of the same jet with cylinders will be shown. These results are representative of the other configurations.

### General flow characteristics with very high stem-generated shear

**Figure 3:**
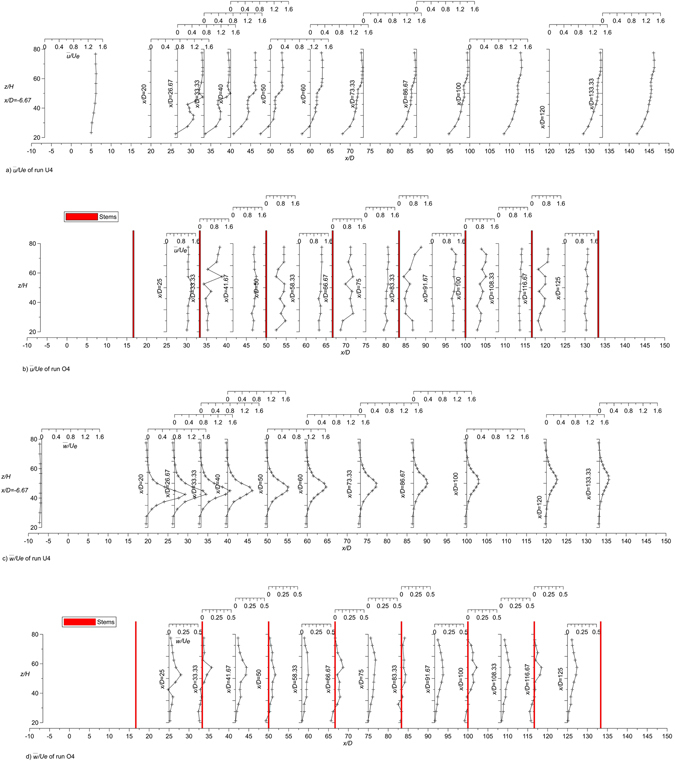
Longitudinal profiles of the dimensionless time-averaged streamwise *u* and vertical *w* velocity components of runs U4 and O4.

**Figure 4:**
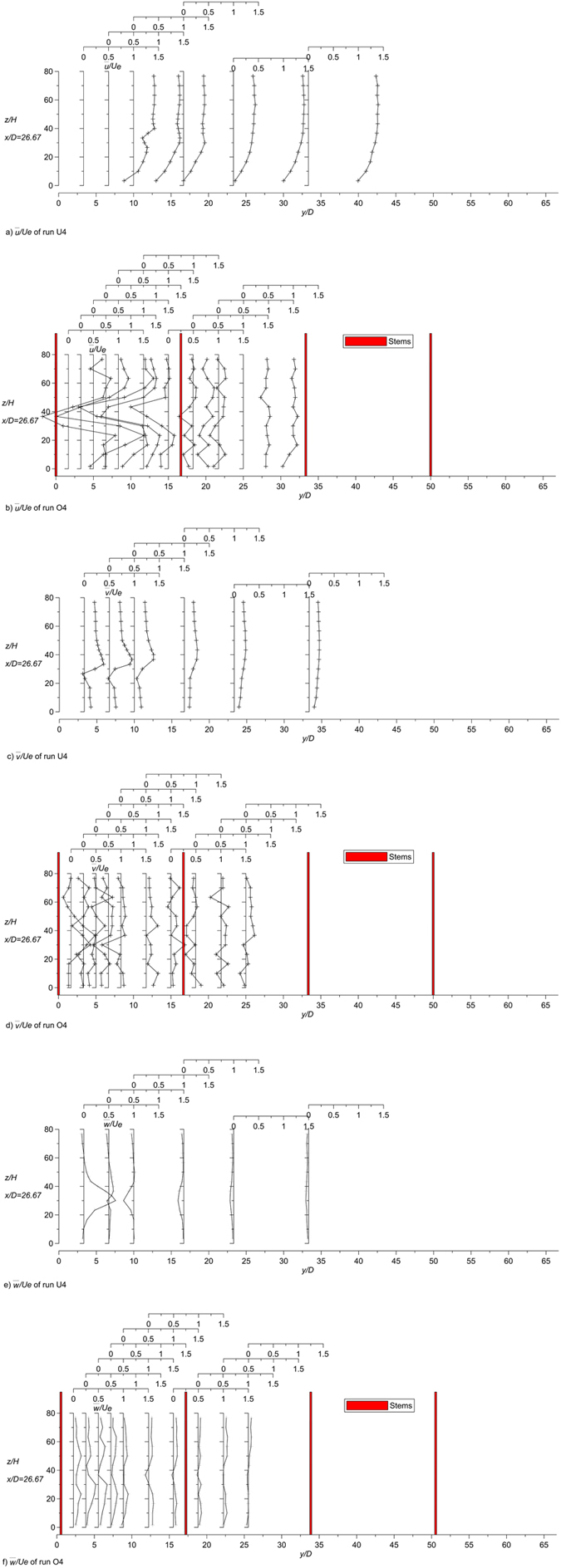
Transversal profiles of the dimensionless time-averaged longitudinal *u*, transversal *v* and vertical *w* velocity components of runs U4 and O4.

Figure 3 shows the longitudinal profiles of dimensionless streamwise $\overline{u}$ and vertical $\overline{w}$ velocity for runs U4 (jet with unvegetated channel current) and O4 (jet with vegetated channel current). The first profile of run U4 is upstream of the jet exit and shows an undisturbed channel flow. The same channel flow is of course present in run O4 and, therefore, is not shown again. Figure 4 shows some transverse profiles of dimensionless streamwise $\overline{u}$, spanwise $\overline{v}$ and vertical $\overline{w}$ velocity components for runs U4 and O4. The velocity fields in Figs 3 and 4 show that the jet spreads more rapidly in the presence of the cylinders.

The complicated nature of the jet in a crossflow is well known in literature (see, for example, Andreopoulos and Rodi^49^). The most obvious feature of the jet in a crossflow is the mutual deflection of both the jet and crossflow. The jet is bent over by the cross-stream, while the latter is deflected, as if it was blocked by a rigid obstacle. The difference being that the jet interacts with the deflected flow and entrains fluid from it. In the case of a very small velocity ratio, generally for *R* = O(1), the flow behaves as if a partial, inclined cover were put over the front of the exit hole, causing the jet streamlines to start bending while still in the discharge tube and the jet to bend over completely right above the exit. The oncoming flow is lifted up over the bent-over jet^49^. In the case of a higher velocity ratio, as in the present study, the jet is only weakly affected near the exit and penetrates the cross-stream before it is bent over, as shown by the velocity fields in Figs 3 and 4. In both cases, wake regions with very complex three-dimensional flow patterns form in the lee of the jet. In these regions, the longitudinal velocity accelerates and the conservation of mass requires a reduction of the transversal velocity components from the sides towards the plane of symmetry. This is shown in the profiles of the transversal velocity of Fig. 4 at the level of the jet. This behavior is emphasized in the runs with cylinders. Very close to the lateral wall a reverse-flow region forms, and cross-stream fluid has been observed to enter this region, travel upstream and then to be lifted upwards by the jet fluid and to be carried downstream together with it. This behavior is clearly shown in the case of run U4 and is still present in the case of run O4, even if the presence of cylinders makes the flow much more complex.

The longitudinal mean velocity $\overline{u}$ varies with the *y*-coordinate (e.g. see *x*/*D* = 26.67 in Fig. 4). In run U4 the $\overline{u}$ velocities at *y*/*D* > 0 are always higher than at the symmetry plane (*y*/*D* = 0) because the wake center with low velocities is at the symmetry plane. Figure 4 shows that starting at *y*/*D* = 0, the $\overline{u}$ velocity can even be seen to increase as the lateral wall is approached. This behavior is due to the deflection of the crossflow around the jet near the lateral wall, which causes an acceleration of the deflected flow. The deflection of the cross-stream around the jet is evident also from the analysis of the $\overline{v}$ velocities, which are oriented towards the wall (Fig. 4). This behavior is enhanced in the presence of the cylinders, because the array causes the jet to spread more rapidly, so that the jet in the array causes greater deflection of the oncoming flow towards the wall. Consequently, the transversal velocities $\overline{v}$ increase more rapidly with distance from *y* = 0.

In run U4, the vertical velocities $\overline{w}$ are larger in the section closest to the symmetry plane, and the highest values in each profile increase with streamwise distance from the nozzle. The same jet issued in the same cross-flow but with the cylinders shows analogous behavior; however, the profiles of the vertical velocities are flatter, i.e. they do not show a significant peak near the jet axis. The approaching flow is also deflected vertically over the jet, and this causes the positive $\overline{w}$-velocity to change sign (in the longitudinal sections farther from the symmetry plane). This behavior is much less evident when the cylinders are present, as shown in the cross section at *x*/*D* = 26.67 of run O4.

**Figure 5:**
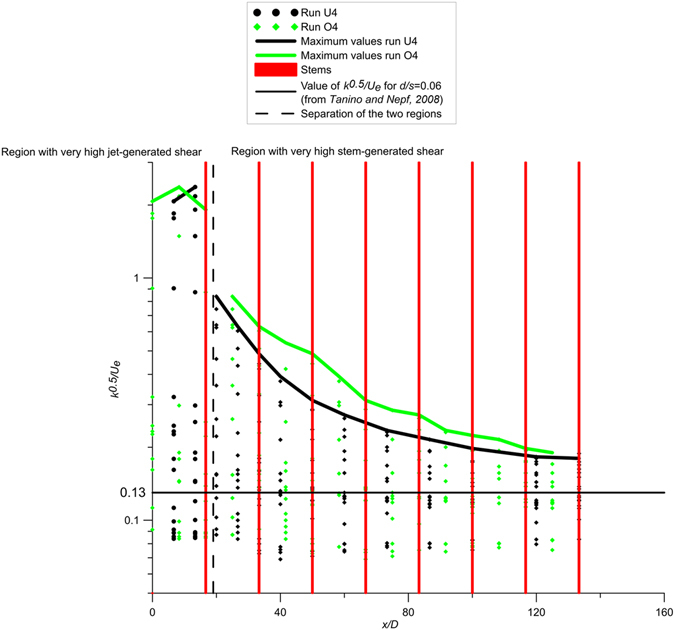
Values of *k* ^*0.5*^/*Ue* with the definition of the region with very high jet-generated turbulence (on the left) and the region with very high-stem generated turbulence (on the right). The figure shows also the expected value of *k* ^*0.5*^/*Ue* for *d/s* = 0.06 for obstructed channel flow (from Tanino and Nepf^30^).

Figure 5 shows the values of $\sqrt{k}$ nondimensionalized by *U*_*e*_ in different regions of runs U4 and O4. Close to the nozzle, specifically *x*/*D* < 19, the values of $\sqrt{k}$ are similar with and without the stem array, indicating that when the lateral length-scale of the jet is small compared to the cylinder spacing, the impact of the cylinder array is small. This trend is also confirmed by the other runs. However, farther from the nozzle, as the length-scale of the jet grows larger, the values of $\sqrt{k}$ are higher with the array, and their average in each vertical section is almost constant. The horizontal dashed line in Fig. 5 shows the value of $\sqrt{k}/{U}_{e}$ predicted from the wake-production model developed by Tanino and Nepf^30^. For the low solid volume fraction, *ϕ* = 0.00283, the drag coefficient, *C*_*D*_ = 1.15, can be estimated from the stem Reynolds number (*Re*_*d*_ = *U*_*e*_ *d*/*ν* = 570) using relations for and isolated cylinder (Tanino and Nepf^9^). With these values, eq 4.1 in Tanino and Nepf^30^ predicts *k* ^0.5^/*U*_*e*_ = 0.13. At large distances from the nozzle (*x*/*D* > 100), where the jet is very dispersed, this prediction agrees well with the average intensity of $\sqrt{k}/{U}_{e}$ measured in the cross-section.

### Integral length scales and turbulent diffusion coefficients

**Figure 6:**
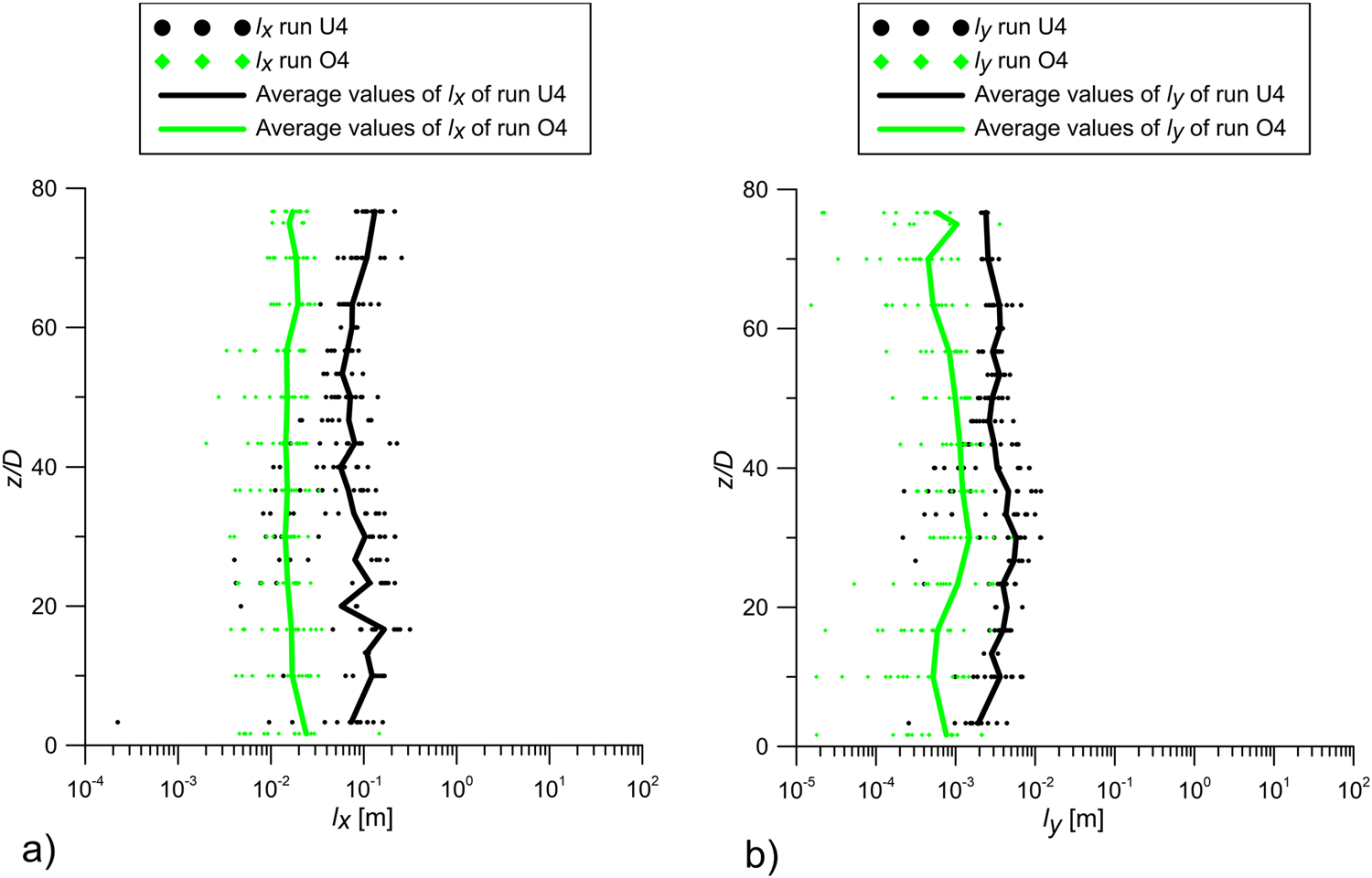
Values of *l*_*x*_ and *l*_*y*_ of runs U4 and O4 with the line of the averaged-values.

Figure 6 compares the integral length-scales with the jet alone (U4, black dots and line) and the jet within the model array of model vegetation (O4, green dots and line). The dots in Fig. 6 represent individual estimates at all *x* positions in the analyzed longitudinal planes of Fig. 3 of runs U4 and O4, respectively, and the lines represent their averages. The average of multiple measurements at the same vertical position are shown with heavy curves. In the unobstructed channel (U4 in Fig. 6), *l*_*x*_ ≈ O(*H*) and *l*_*y*_ ≈ O(*b*). The presence of the cylinder array reduces the length-scales in both the streamwise (*l*_*x*_) and cross-stream (*l*_*y*_) directions. Specifically, in the presence of the array (O4 in Fig. 6), *l*_*x*_ ≈ 0.02 m, which is on the order of the stem spacing *s*, and *l*_*y*_ ≈ 0.001 m, which is on the order of the stem diameter *d*. The reduction in turbulence length-scales is consistent with the domain geometry, i.e., the cylinder diameter, *d*, and spacing, *s*, are much smaller than the jet width, *b*, and flow depth, *H*. The array introduces turbulence at the scale of the stem diameter *d* and spacing *s*, and it breaks apart the larger scales of turbulence associated with the jet (width *b*) and channel depth (*H*).

**Figure 7:**
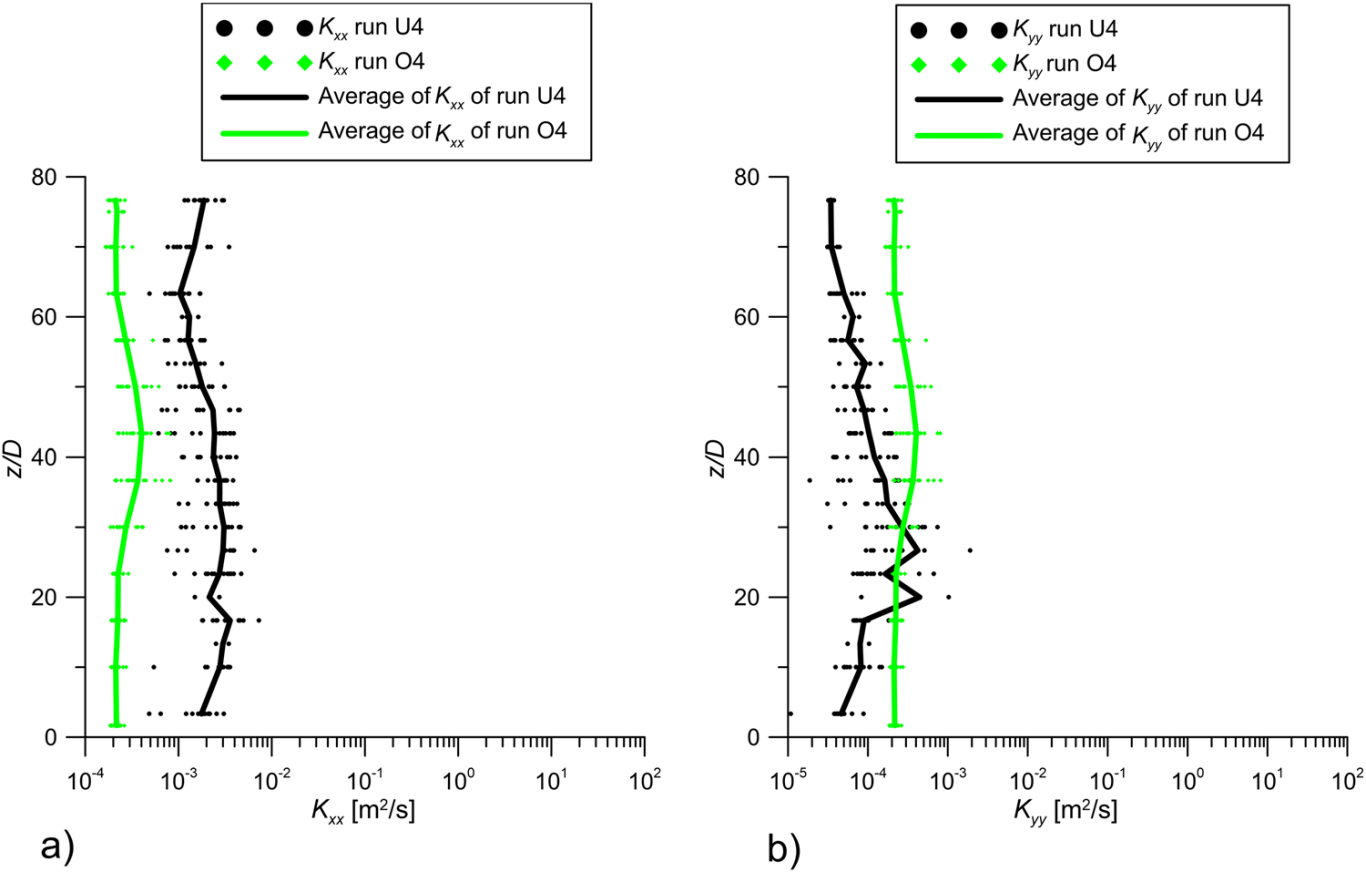
Values of *K*_*xx*_ and *K*_*yy*_ of runs U4 and O4 with the line of the averaged-values.

Figure 7a and b show the vertical profile of streamwise, *K*_*xx*_, and transverse, *K*_*yy*_, turbulent diffusion coefficient for runs U4 and O4, estimated from eq. (4). The dots in Fig. 7 represent individual estimates at all *x* positions in the analyzed longitudinal planes of Fig. 3 of runs U4 and O4, respectively, and the lines represent their averages. In the case with the unobstructed jet (U4), the streamwise diffusivity (*K*_*xx*_ ≈ 0.003 m^2^s^−1^) is larger than the transverse diffusivity (*K*_*yy*_ ≈ 10^−4^m^2^ s^−1^) The observed trends are totally different when the jet flows within the cylinder array (O4). First, in the obstructed condition the streamwise and transverse diffusivity have the same magnitude (*K*_*xx*_ = *K*_*yy*_ = 2 × 10^−4^ m^2^s^−1^). Further, the streamwise diffusion coefficient *K*_*xx*_ is significantly reduced in comparison to the unobstructed condition. In contrast, the transverse turbulent diffusion coefficient *K*_*yy*_ is enhanced by the array obstruction (O4), relative to the jet without obstruction (U4), confirming the theoretical results on the jet detrainment process briefly described in the theoretical framework of the present paper and deeply analyzed and demonstrated by Mossa and De Serio^31^. In fact, in the case of obstructed flows, jets experience a detrainment process, with which the jet fluid enters the ambient fluid. This result is consistent with the increase of the transverse diffusion coefficient of run O4, shown in Fig. 7, which reveals a diffusion from the jet axis towards the ambient. Finally, the average of the experimental values of *K*_*yy*_ ( = 2 × 10^−4^ m^2^s^−1^) in the obstructed flow is in good agreement with the model developed in Tanino and Nepf^30^. Specifically, for solid volume fraction *ϕ* = 0.00283 (present study), eq. 2.16 and 4.1 in Tanino and Nepf^30^ predict *K*_*yy*_ = 0.18*U*_*e*_ *d* = 1 × 10^−4^ m^2^ s^−1^. This agreement suggests that within the array, the turbulent diffusion is dominated by array-generated turbulence, with little influence from jet-generated turbulence.

### Advection terms

**Figure 8:**
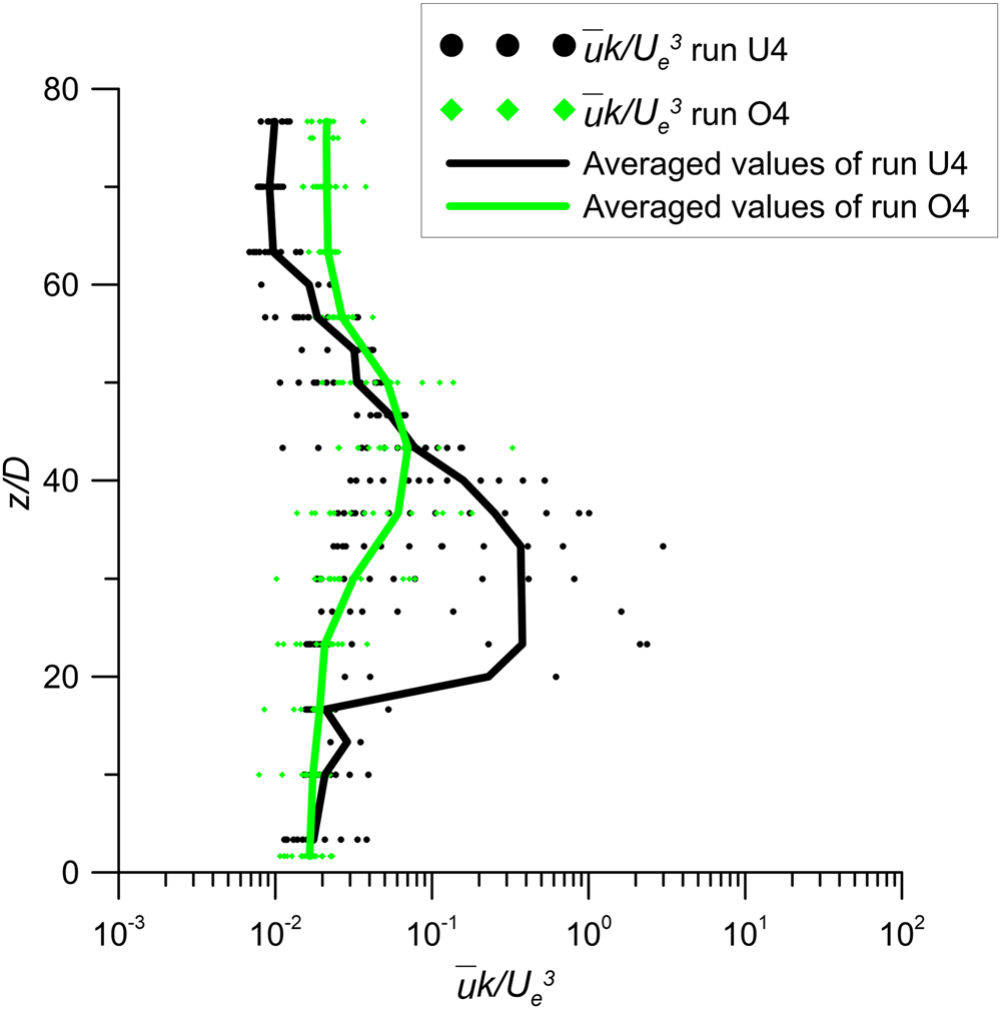
Values of $\overline{u}k$ of runs U4 and O4 of the longitudinal section with the line of the averaged-values.

**Figure 9:**
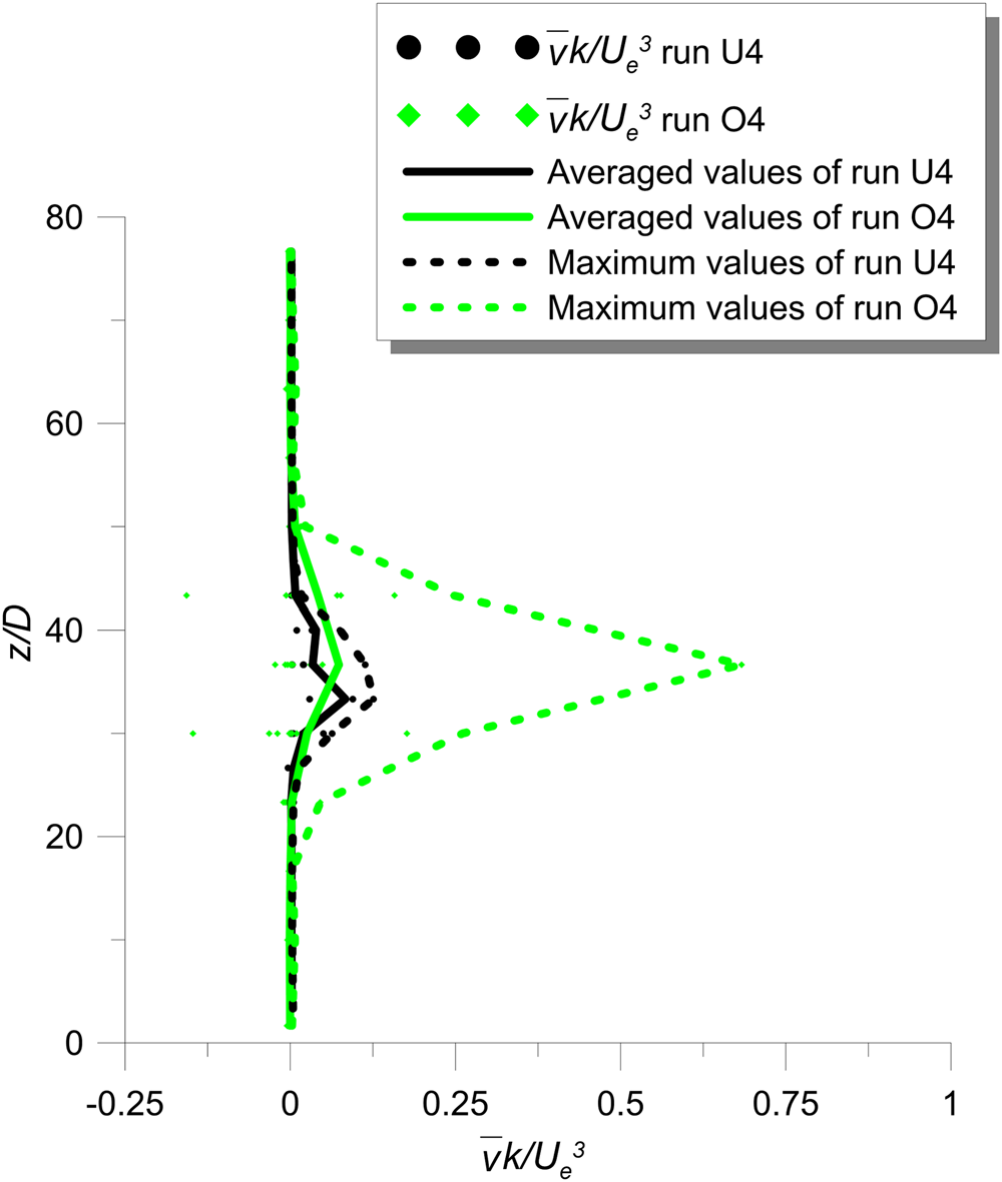
Values of $\overline{v}k$ of runs U4 and O4 of the transversal section at *x*/*D* = 26.67 with the lines of the averaged-values and the maximum-values.

Figure 8 shows the vertical profiles of $\overline{u}k$ of the runs U4 and O4. The dots in Fig. 8 represent individual estimates at all *x* positions in the analyzed longitudinal planes of Fig. 3 of runs U4 and O4, respectively, with the lines of the averages. Figure 9 shows the values of $\overline{v}k$ of runs U4 and O4, where the dots represent individual estimates at all *y* positions in the analyzed transversal at *x*/*D* = 26.67 with the lines of the averages and the maxima.

The averaged values of Fig. 8 demonstrate that the streamwise advection of the jet in the unobstructed flow is greater than that of the jet in the obstructed flow. In contrast, the averaged and maximum values shown in Fig. 9 demonstrate that the transverse advection increase with the stems, due to the deflection of the longitudinal flow towards the lateral direction. These results are better shown in Figs 8 and 9 with the lines of averaged values, which enable us to quantify the difference between the cases with and without the array.

From the experimental results presented above, it is possible to conclude that the presence of cylinders reduces both the diffusion and advection processes in the longitudinal direction. In contrast, the lateral dispersion does not experience the same reduction, because of the lateral deviation of the streamwise flow around individual cylinders.

### Estimation of dissipation rate

**Figure 10:**
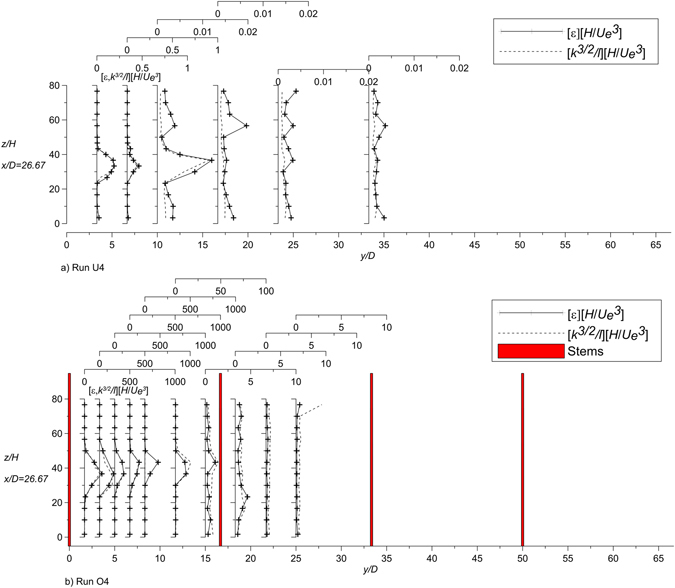
Estimated and observed dissipation rate of runs U4 and O4 at the transversal section *x*/*D* = 26.67.

**Figure 11:**
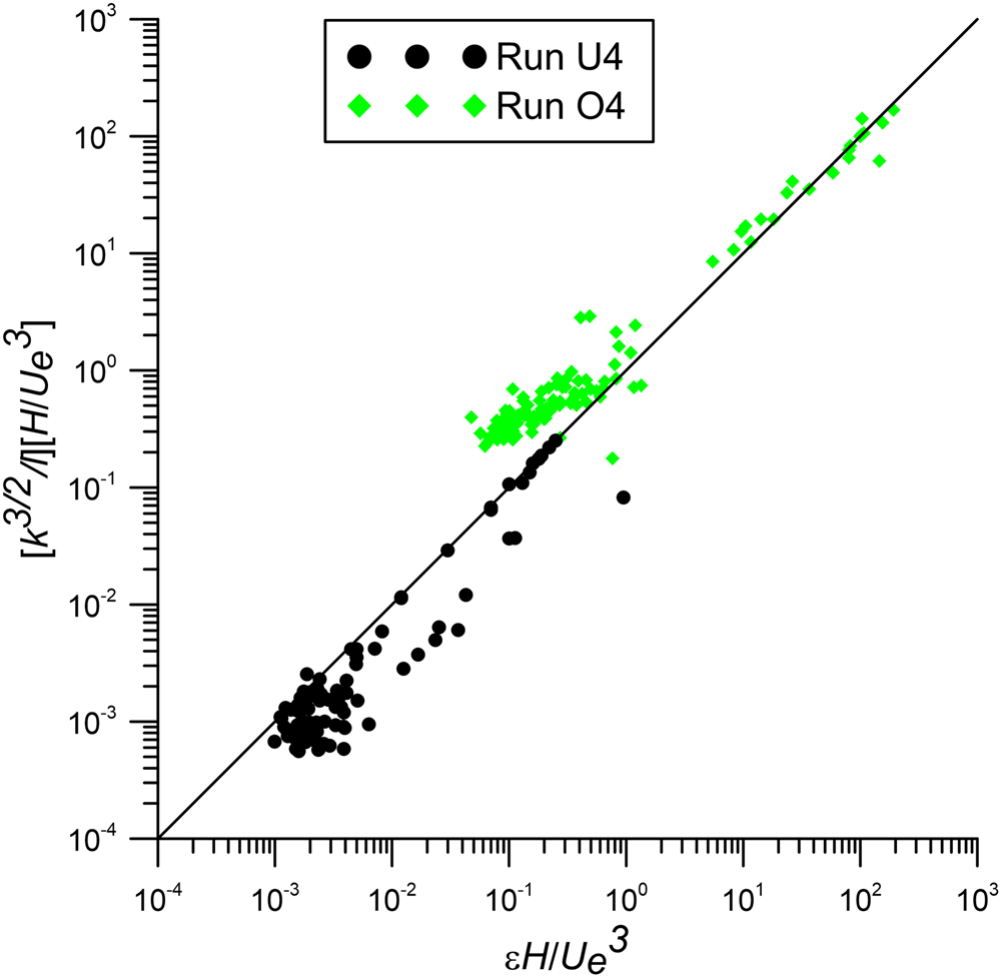
Estimates of dissipation rate of the cross section at *x*/*D* = 26.67 from the jet nozzle (based on the scaling *l*~O(*H*) for run U4 and on *l*~O(*d*) for run O4) with the observed dissipation rate *ε*.

Figure 10a compares the vertical profiles of estimated dissipation rate based on the scaling *l*~O(*H*) and the observed dissipation rate values *ε*. Similarly, in Fig. 10b the observed dissipation rate is compared to the estimated value based on the scaling *l*~O(*d*)^50^ for the obstructed case (run O4). Specifically, for the cross section at *x*/*D* = 26.67 the comparison between the estimated and observed dissipation rates is plotted in Fig. 11. Figs 10 and 11 confirm that *ε* scales with *k* and *l*, as shown in paragraph 2.2.

**Figure 12:**
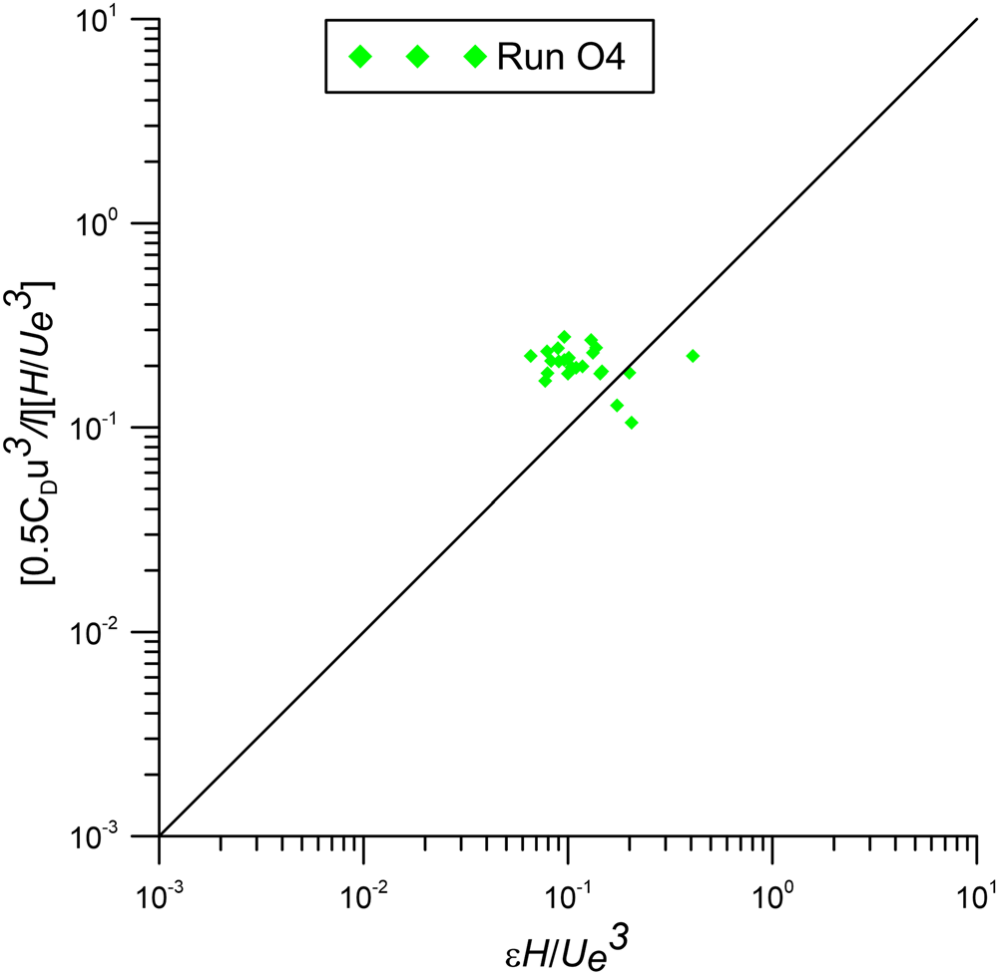
Estimates of dissipation rate of the cross section at *x*/*D* = 26.67 from the jet nozzle compared with the wake production in the zone of the interface of jets and channel flow.

**Figure 13:**
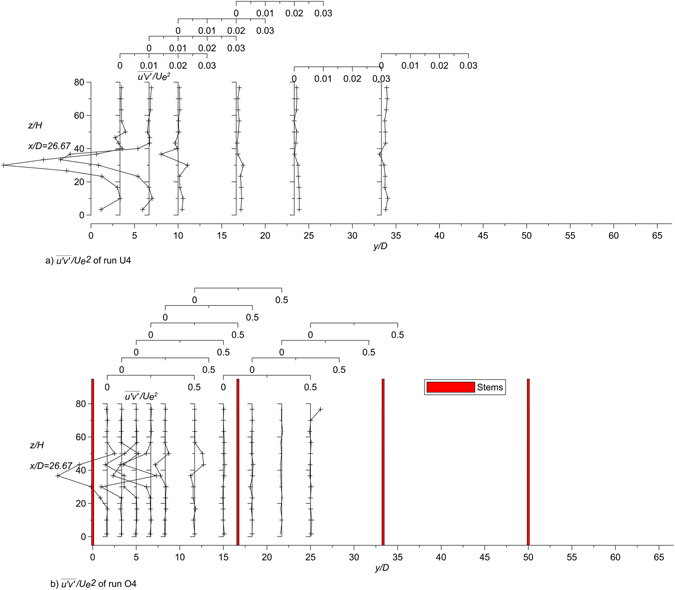
Transversal profiles of the dimensionless time-averaged values of u’v’ of runs U4 and O4.

For run O4, Fig. 12 shows a comparison between the measured turbulent energy dissipation and the wake production estimated by the last term of the RHS of eq. (9) using *C*_*D*_, *a* and $\overline{u}$. The figure shows only the measurement points close to the interface of the jet and the channel flow. In fact, Fig. 13 demonstrates that, at the interface between the cross-section of the jet and the channel flow, the shear terms $\overline{u^{\prime} v^{\prime} }$ become very small. This behavior confirms that at the interface between the jet cross-section and the channel flow, the production term of the turbulent energy is dominated by the wake formation around the cylinders and is balanced by the viscous dissipation. Therefore, Fig. 12 confirms the theoretical analysis of eq. (8) for the external region of the jet cross-sections in obstructed flows.

Since the values of the turbulent energy dissipation and production are closely related with the large-scale eddies^38^, it is possible to conclude that the vegetation play a crucial role in mass and momentum exchange.

## Conclusions

Turbulent jets flowing in currents have been widely examined because of their relevance to many environmental conditions. This study examines a pure turbulent jet issued into an obstructed flow, simulated with a regular array of cylinders. The main conclusions can be summarized as follows:

1. Differently from the case of jets in unobstructed flows, in the presence of a cylinder array, the streamwise turbulent diffusion is reduced, while the transverse diffusion is enhanced. Importantly, in the obstructed condition, the streamwise and transverse turbulent diffusion coefficients are of the same order of magnitude.
2. The presence of the cylinder array reduces both the diffusion and advection processes of the jet in the longitudinal direction. In contrast, the lateral dispersion does not experience the same reduction, because of the transversal deviation of the streamwise flow around individual cylinders.
3. At the interface between the cross-section of the jet and the channel flow, the Reynolds shear stresses are very small. Consequently, the production term of the turbulent energy, prevalently due to the wake formation around the cylinders, is balanced by the viscous dissipation. This behavior confirms eq. (9).

## Electronic supplementary material

Supplementary Information
