# Supplementary material for: How vegetation in flows modifies the turbulent mixing and spreading of jets
Source: Sci Rep. 2017 Jul 26;7:6587. doi: 10.1038/s41598-017-05881-1 (PMC5529552; doi:10.1038/s41598-017-05881-1)
Supplement: Supplementary file 1 — Supplementary Information [file 41598_2017_5881_MOESM1_ESM.pdf]

APPENDIX 1

HOW VEGETATION IN FLOWS MODIFIES THE TURBULENT MIXING AND SPREADING OF JETS

Michele MOSSA[(1)], Mouldi BEN MEFTAH[(1)], Francesca DE SERIO[(1)], Heidi M. NEPF[(2)]*

(1) Department of Civil, Environmental, Land, Building Engineering and Chemistry, Polytechnic University of Bari, Bari, Italy
(2) Department of Civil and Environmental Engineering, Massachusetts Institute of Technology, Cambridge, MA, USA.
(*) Corresponding author, e-mail: michele.mossa@poliba.it

POSSIBLE CASES OF JETS IN OBSTRUCTED FLOWS

In the case of jets in obstructed flows, the type of flow depends strongly on the stem diameter, the jet cross section length scale and the distance between the stems. Particularly, in the present analysis, the scale of the jet is the dimension of its cross-section *b*, generally assumed as the distance between the jet axis, where the longitudinal mean velocity is $\bar{u}_m$ and the point where the mean longitudinal velocity is $\bar{u}$ with $\bar{u}/\bar{u}_m = 1/e$. The other scales are the stem diameter *d* and the distance between the cylinders *s*. The ratios between these scales are *d/s* and *b/d*. From these two dimensionless variables, it is possible to derive the third as their product *b/s*. Considering the above-mentioned three dimensionless parameters, it is possible to classify different jet-type flows as shown in Table A1. Table A1 refers to a regular array of cylinders, but the conclusions are similar when considering random cylinder arrays using other key geometric parameters.

For example, White and Nepf[27] necessarily limited their study considering a two-dimensional, infinite in extent array of cylinders. In that case the array contained cylinders of constant diameter *d* distributed randomly with a constant density number, i.e. the probability density function for finding a cylinder centered at a location is uniform. We can conclude that the type of obstruction is fundamental and surely could change the results. In any case, as already written, the flow classification shown hereafter refers to a regular cylinder array, which is generally one of the most analyzed configurations in literature.

Furthermore, Table A1 briefly explains some effects of the stems on the jet-type flow and ambient current, showing also examples of the flows. The jet-type flows of Table A1 are generally the vehicle of a solute and, therefore, the turbulence and dispersion analysis of this kind of flow, considering a comparison of the cases of an obstructed or an unobstructed ambient current are definitively of interest. The runs analyzed in the present paper are described by the case 2 of Table A1.

**Direct effects on the jet and on the current / Example of flows**	**Sketch of the flow (top view)**
Case 1<br><br>*d/s*=O(1), *b/d*=O(1), *b/s*=O(1)<br><br>Effects mainly on the ambient current and local effects on the jet boundary at the same scale of *b*. Example of flows: peculiar situation of the cases described below.	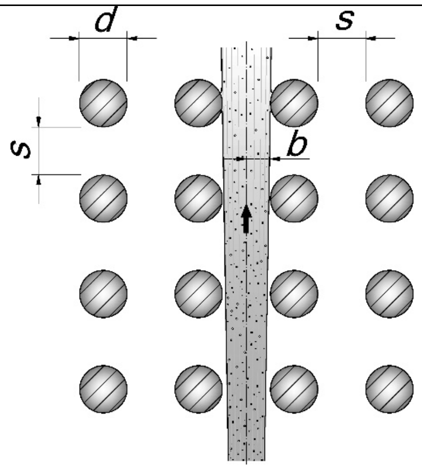
Case 2<br><br>*d/s*=O(≤10^{-1}-1), *b/d*=O(10-10^{2}), *b/s*=O(10-10^{2})<br><br>Widespread effects on both the ambient current and the jet. Example of flows: jets in rivers and sea with vegetated bottom current.	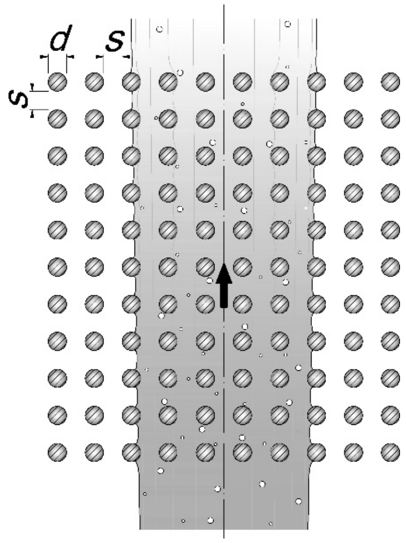
Case 3<br><br>*d/s*= O(10^{-2}-10^{-1}), *b/d*=O(1-10), *b/s*=O(10^{-2}-10^{-1})<br><br>Effects almost absent on the jet and locally present on the ambient current. Example of flows: release of water of boats between oyster farms.	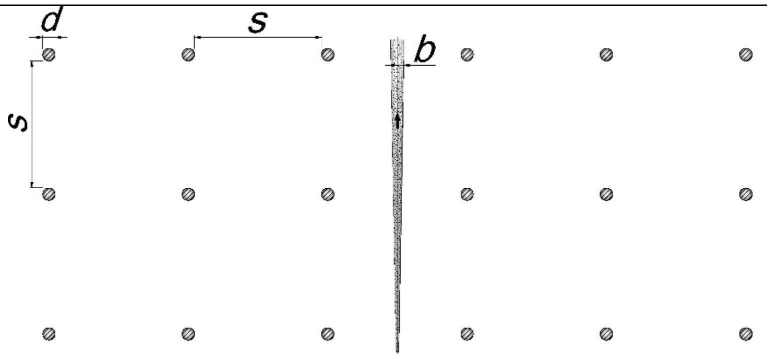

Case 4<br><br>$d/s=O(10^{-2}\text{-}10^{-1})$, $b/d=O(10\text{-}10^{2})$, $b/s=O(1)$<br><br>Local effects on the jet boundary at a scale with an order of magnitude less than *b*. Effects locally present on the ambient current. Example of flows: peculiar situation of the previous case; river in a flood plain with an array of trees.	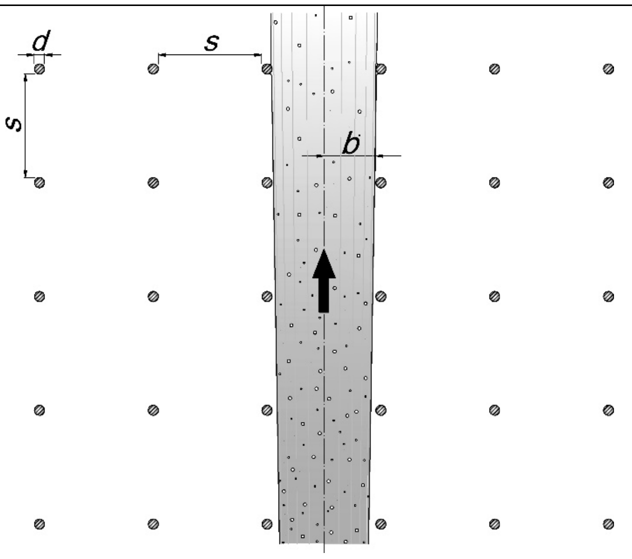
Case 5<br><br>$d/s=O(10\text{-}100)$, $b/d=O(10^{-2}\text{-}10^{-1})$, $b/s=O(1)$<br><br>Very strong effects on the ambient current and locally at the jet boundary. Example of flows: narrow jets in groundwater; release of a dye trace in groundwater.	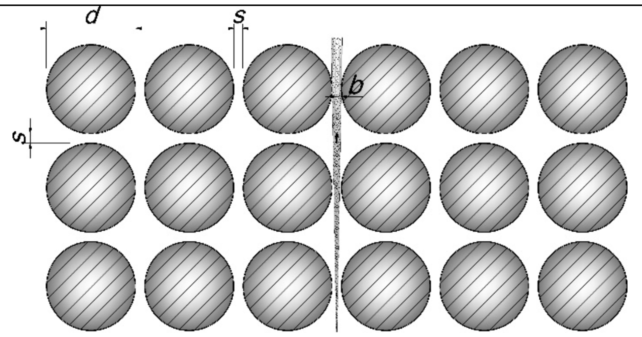
Case 6<br><br>$d/s=O(1)$, $b/d=O(10^{-1})$, $b/s=O(10^{-1})$<br><br>Strong effect on both the jet and the current. Example of flows: Narrows jets in the lower part of oyster farms.	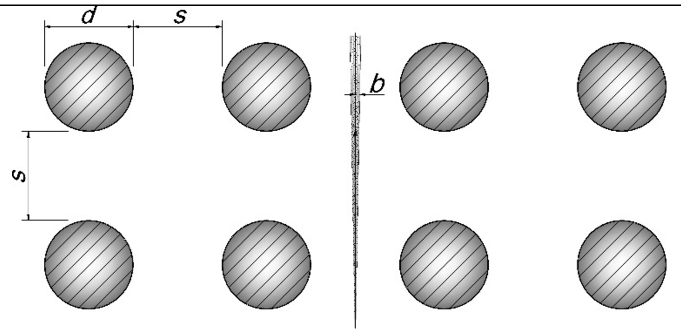
Case 7<br><br>$d/s=O(1\text{-}10)$, $b/d=O(10^{-1}\text{-}1)$, $b/s=O(10\text{-}10^{2})$<br><br>Big effects on both the jet and the ambient current. Example of flows: High-flow-blockage canopy; diffusion of contaminants in porous groundwater; injections of jets in groundwater; interior flows associated with porous obstruction. In this case, the jet will tend to diverge significantly in the canopy.	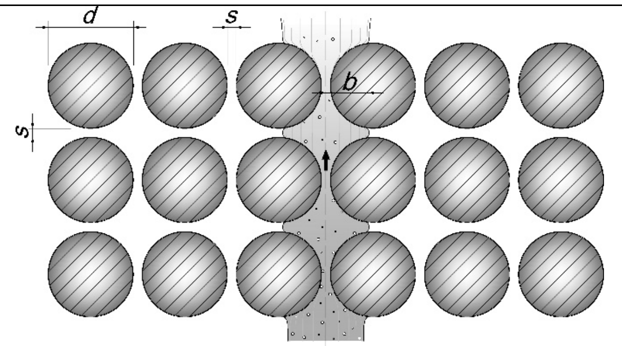

Table A1. Possible cases.
